# An extremely poor nutritional condition enables efficient white cell mating in *Candida albicans*

**DOI:** 10.1128/msphere.00291-25

**Published:** 2025-06-05

**Authors:** Chaoran Zhong, Shuyun Guan, Ming Xu, Mingyang Ma, Chao Li, Li Tao, Guanghua Huang, Ming Guan

**Affiliations:** 1 Shanghai Institute of Infectious Disease and Biosecurity, State Key Laboratory of Genetic Engineering, School of Life Sciences, Department of Laboratory Medicine, Department of Infectious Diseases, Huashan Hospital, Fudan University 12478, Shanghai, China; 2 Department of Laboratory Medicine, Huashan Hospital, Shanghai Medical College, Fudan University 12478, Shanghai, Shanghai, China; Kyungpook National University, Daegu, South Korea

**Keywords:** nutrient depletion, phenotypic switching, opaque, mating, ROS, sexual reproduction, *C. albicans*, cell death

## Abstract

Pathogenic fungi employ diverse strategies to undergo sexual reproduction. It was previously believed that the major human fungal pathogen *Candida albicans* must switch from the “sterile” white state to the mating-competent opaque state to mate. We recently reported that glucose depletion could induce *C. albicans* white cell mating at a moderate frequency. In this study, we demonstrate that white cells can undergo efficient mating when incubated on agar medium (water and agar, without additional nutritional components). Cell survival assays revealed that a subpopulation of white cells underwent cell death, releasing nutritional components that supported surviving cells in undergoing morphological changes and efficient mating. Inactivation of *CST20*, *MAC1*, *MCU1*, and *IDP2*—genes required for cell death and reactive oxygen species generation in *C. albicans*—resulted in a significant reduction in mating frequency under nutrient-depleted conditions. Taken together, our findings suggest that extremely poor nutritional conditions are able to promote epigenetic switch-independent mating in *C. albicans*.

**IMPORTANCE:**

By demonstrating that white cells can mate under nutrient-depleted conditions, the research uncovers a novel mechanism of sexual reproduction in this pathogenic fungus. The findings suggest that cell death, through the release of nutritional components and the generation of reactive oxygen species, plays a crucial role in facilitating mating under nutrient-depleted conditions. This research not only provides novel insights into the reproductive strategies of *C. albicans* but also highlights potential avenues for exploring altruistic behaviors in other microorganisms. Understanding these mechanisms could have significant implications for the development of new therapeutic strategies to combat fungal infections, particularly in environments where nutrient limitations are common.

## INTRODUCTION

Sexual reproduction is widespread in fungi and acts as a powerful evolutionary driver for the development of new traits and genetic diversity (1–4). Different fungal species employ diverse strategies to undergo sexual reproduction (5–8). To achieve efficient mating, the major human fungal pathogen *Candida albicans* and its closely related species, such as *Candida tropicalis* and *Candida dubliniensis*, must first switch from the “sterile” white state to the mating-competent opaque state (9–11). The white and opaque cell types of *C. albicans* differ not only in their mating competency but also in other biological features, including morphological appearance, metabolism, global gene expression profiles, and virulence (9, 12–14). This epigenetic switch serves as an additional layer of regulation for sexual mating in these pathogenic fungi.

In natural settings, the “mating-competent” opaque cell type likely constitutes a minority, while white cells represent the default state of *C. albicans* (13, 15). We previously reported that white and opaque cells can coordinate within a mixed culture system to promote mating (16). White cells are capable of expressing and secreting sexual pheromones, creating an environment conducive to opaque cell mating (16). Given the high cost of sexual reproduction, this coordination between the two cell types may represent a trade-off strategy between sexual and asexual lifestyles in *C. albicans*. Additionally, we recently discovered that glucose depletion induces white cell mating at a moderate frequency by activating the pheromone-sensing and mating-associated MAPK pathway (17). This finding suggests that under specific conditions, such as those encountered in natural niches, white cells may exhibit mating competency. Moreover, glucose starvation induces efficient same-sex mating in *C. albicans* owing to the accumulation of intracellular reactive oxygen species (ROS), which overpower the Hsf1-Hsp90 pathway (18). Atmospheric relative humidity (RH) has also been identified as a regulator of *C. albicans* same-sex mating, with low RH conditions favoring mating (19). These observations indicate that *C. albicans* employs multiple strategies to achieve sexual reproduction.

In this study, we aimed to investigate the environmental factors and mechanisms underlying efficient white cell mating in *C. albicans*, based on our previous observations. We demonstrate that complete nutrient depletion (agar medium without nutritional components) induces efficient white cell mating in a manner independent of the white-opaque phenotypic switch. Under these extreme nutrient-poor conditions, a subpopulation of *C. albicans* white cells undergoes cell death, releasing essential nutrients that support the growth and mating of surviving cells. Consistently, the inactivation of Cst20 (kinase), Mac1 (transcription factor), and mitochondrial metabolism-related proteins required for cell death under stress significantly reduced the frequency of white cell mating. These findings indicate that white cells of *C. albicans* are capable of undergoing efficient mating under certain conditions.

## RESULTS

### Extremely poor nutritional conditions promote efficient white cell mating in *C. albicans*

Nutrient availability is a critical regulator of fungal sexual reproduction (20, 21). In a previous study, we observed that the depletion of carbon sources and other nutrient components promotes white cell mating in *C. albicans *(17, 18). Notably, preliminary results indicated that white cells could undergo efficient mating under extreme nutrient-poor conditions (2% agar + K_2_HPO_4_) (17). In the present study, we aimed to determine the optimal culture conditions for white cell mating and to investigate underlying mechanisms.

**Fig 1:**
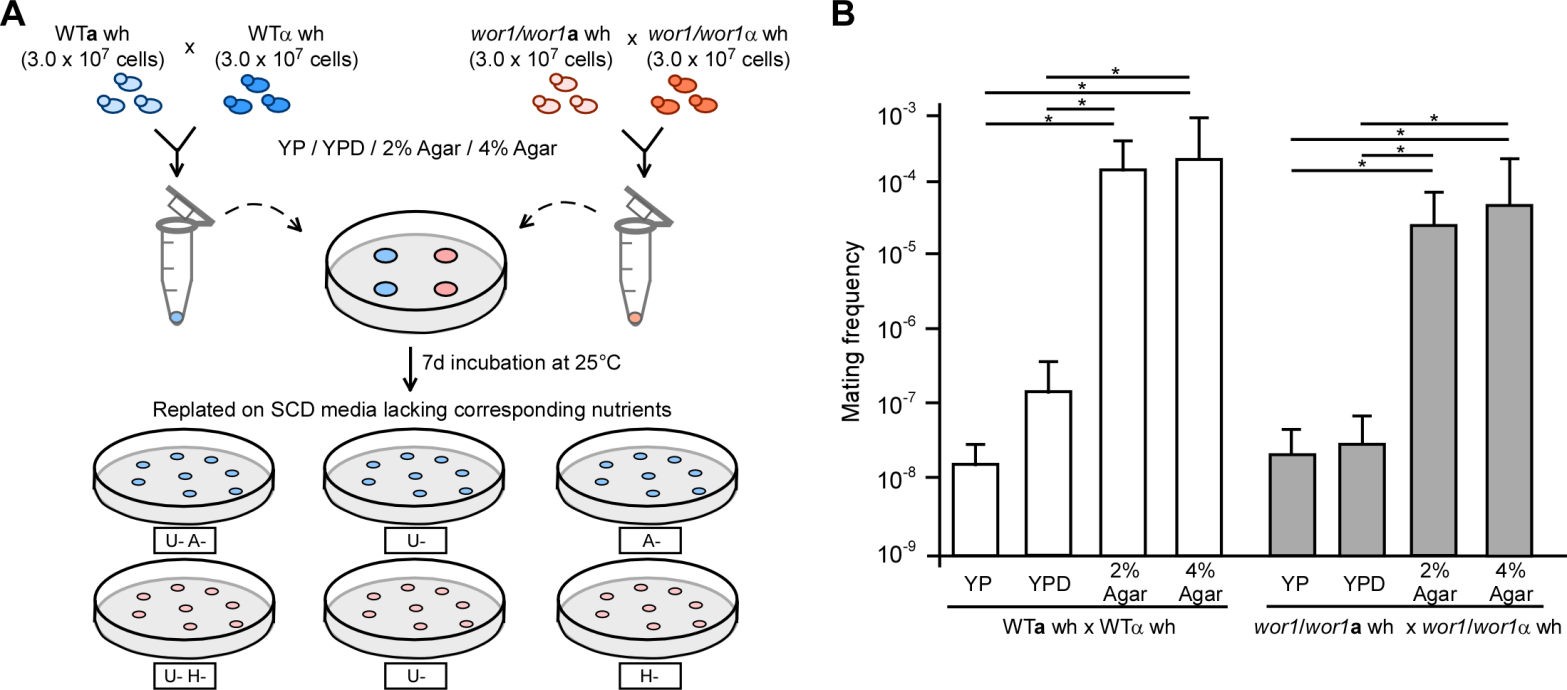
Nutrient-depleted conditions enable efficient white cell mating in *C. albicans*. (**A**) Schematic illustration of quantitative mating assays. White cells of the **a** and α mating partners (3 × 10^7^ cells for each) were mixed, spotted onto different medium plates, and incubated at 25°C under 25% relative humidity (RH). YP, YPD, 2% agar, and 4% agar media were examined. The 2% agar and 4% agar media contained only ddH_2_O and agar (2% and 4%, respectively) and did not contain any other nutrient components. After 7 days of incubation, the mating mixtures were plated onto synthetic complete dextrose (SCD) media lacking corresponding nutrients to determine mating frequencies. wh, white cells. (**B**) Mating frequencies of the WT**a** × WTα and *wor1/wor1***a** × *wor1/wor1*α crosses on different media. Strains used: WT**a**, LTS1024; WTα, GH1710; *wor1/wor1***a**, GH1248; and *wor1/wor1*α, CAY3336. Three independent experiments were performed. The result represents the average ±standard deviation (SD). Statistical differences were determined by two-sided unpaired Student’s *t*-test. * indicates *P* < 0.05. This figure is associated with Table S1. Detailed strain information is included in the supplemental material (Table S4).

Mating mixtures of the wild-type (WT) cross (WT**a** wh × WTα wh) were cultured on 2% agar and 4% agar media (water + 2% or 4% agar, without additional nutrients) under 25% atmospheric RH (Fig. 1A). Glucose-depleted YP medium and nutrient-rich YPD medium (without K_2_HPO_4_ [17]) were used as controls. After 7 days of incubation at 25°C, mating mixtures were resuspended in ddH_2_O, diluted, and plated on selectable synthetic complete dextrose (SCD) medium for auxotrophic growth analysis. As shown in Fig. 1B and Table S1, the mating frequencies of WT white cell crosses on 2% and 4% agar media were approximately 1,000- to 10,000-fold higher than those observed on YPD or YP media.

To rule out the possibility that white cells of the WT strains might switch to the mating-competent opaque form, thereby increasing mating frequency, we first assessed the white-to-opaque switching frequencies of WT strains. As shown in Table S2, the switching frequencies were very low on agar media. Subsequently, we conducted quantitative mating assays using white phase-locked *wor1/wor1* mutant strains under the same culture conditions. As shown in Fig. 1B and Table S1, the mating frequencies of the *wor1/wor1***a** wh × *wor1/wor1*α wh cross on agar media were comparable to those observed for the WT cross. These results indicate that extreme nutrient-poor conditions promote efficient white cell mating in *C. albicans*.

### Elevated transcriptional expression of mating-associated genes under nutrient-depleted conditions

**Fig 2:**
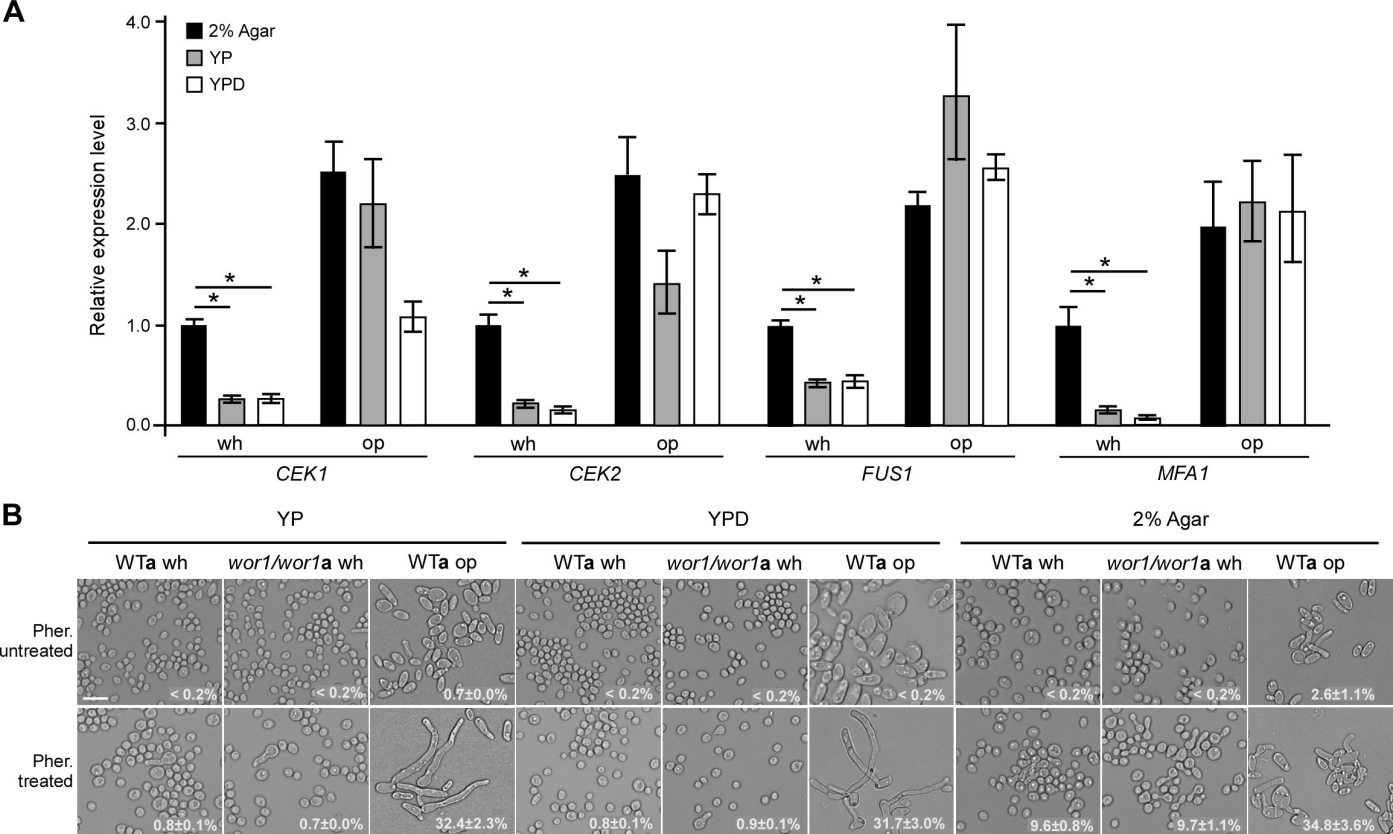
Nutrient depletion promotes the transcriptional expression of mating-associated genes (**A**) and sensitizes white cells to pheromone (**B**). (**A**) Approximately 3 × 10^7^ cells of the *wor1/wor1***a** white (GH1248) and WT**a** opaque (LTS1024) cells were spotted onto different media and incubated at 25°C under 25% RH for 5 days. Statistical differences were determined by two-sided unpaired Student’s *t*-test. *, *P* < 0.05. Scale bar: 10 µm. *CEK1*, *C. albicans* ERK-like kinase; *CEK2*, *C. albicans* extracellular signal-regulated kinase; *FUS1*, cell fusion 1; *MFA1*, mating type A1. (**B**) Approximately 1.5 × 10^7^ of white or opaque cells were spotted onto different media and incubated at 25°C under 25% RH for 7 days. Three microliters of 200 µM α-pheromone was added onto the spots every 24 h (at 24, 48, 72, 96, and 120 h). One representative image of three independent experiments is shown. Percentages of cells with mating projections (average ±SD) are indicated in the corresponding images. Strains used: WT**a**, LTS1024; *wor1/wor1***a**, GH1248. Pher, α-pheromone. wh: white, op: opaque. Scale bar: 10 µm.

To determine the distinct mating responses under different culture conditions, we examined the relative expression levels of four mating-related genes (*FUS1*, *MFA1*, *CEK1*, and *CEK2*) in *wor1/wor1* white cells. *FUS1* encodes a membrane protein essential for cell fusion and efficient mating, *MFA1* encodes the **a**-pheromone precursor, and *CEK1* and *CEK2* encode MAP kinases that regulate mating (22, 23). As shown in Fig. 2A, the relative expression levels of these genes in white cells were significantly higher on 2% agar medium than on YP and YPD media. The relative expression levels of these genes in opaque cells were much higher than those in white cells. These results suggest that the increased expression of mating-associated genes under nutrient-depleted conditions may contribute to the efficient mating of *C. albicans* white cells.

To determine whether white cells exhibit an enhanced response to pheromone under nutrient-depleted conditions, we treated white and opaque cells of both the WT and *wor1/wor1 MTL***a** mutant strains with α-pheromone. As shown in Fig. 2B, no projections were observed in the white cells of the WT and *wor1/wor1* mutant strains in the absence of α-pheromone. However, a subset of white cells in both strains developed clear mating projections when treated with α-pheromone on 2% agar medium. In contrast, when grown on YP and YPD media, white cells formed very few mating projections, regardless of α-pheromone presence. Opaque cells of the WT strain, serving as a positive control, exhibited robust mating projections on all three media when treated with pheromone. Overall, these findings indicate that nutrient depletion promotes pheromone responses and mating in *C. albicans* white cells.

Notably, the mating frequency of *C. albicans* white cells on YP + K (YP plus 0.25% K_2_HPO_4_) medium was previously reported (17). Here, we compared mating-related gene expression and mating frequencies of WT white cells grown on YP and YP + K media (17). As shown in Fig. S1, the relative expression levels of *FUS1*, *MFA1*, *CEK1*, and *CEK2* genes in white cells grown on YP + K medium were significantly higher than those on YP medium. Correspondingly, the mating frequency of the WT cross was increased 1,978-fold on YP-K medium relative to that on YP medium. This difference could be attributed to the presence of K_2_HPO_4_ in the YP + K medium, which buffered the medium pH and thus facilitated mating in *C. albicans*.

### Nutrient depletion induces cell death in a subset of white cells

**Fig 3:**
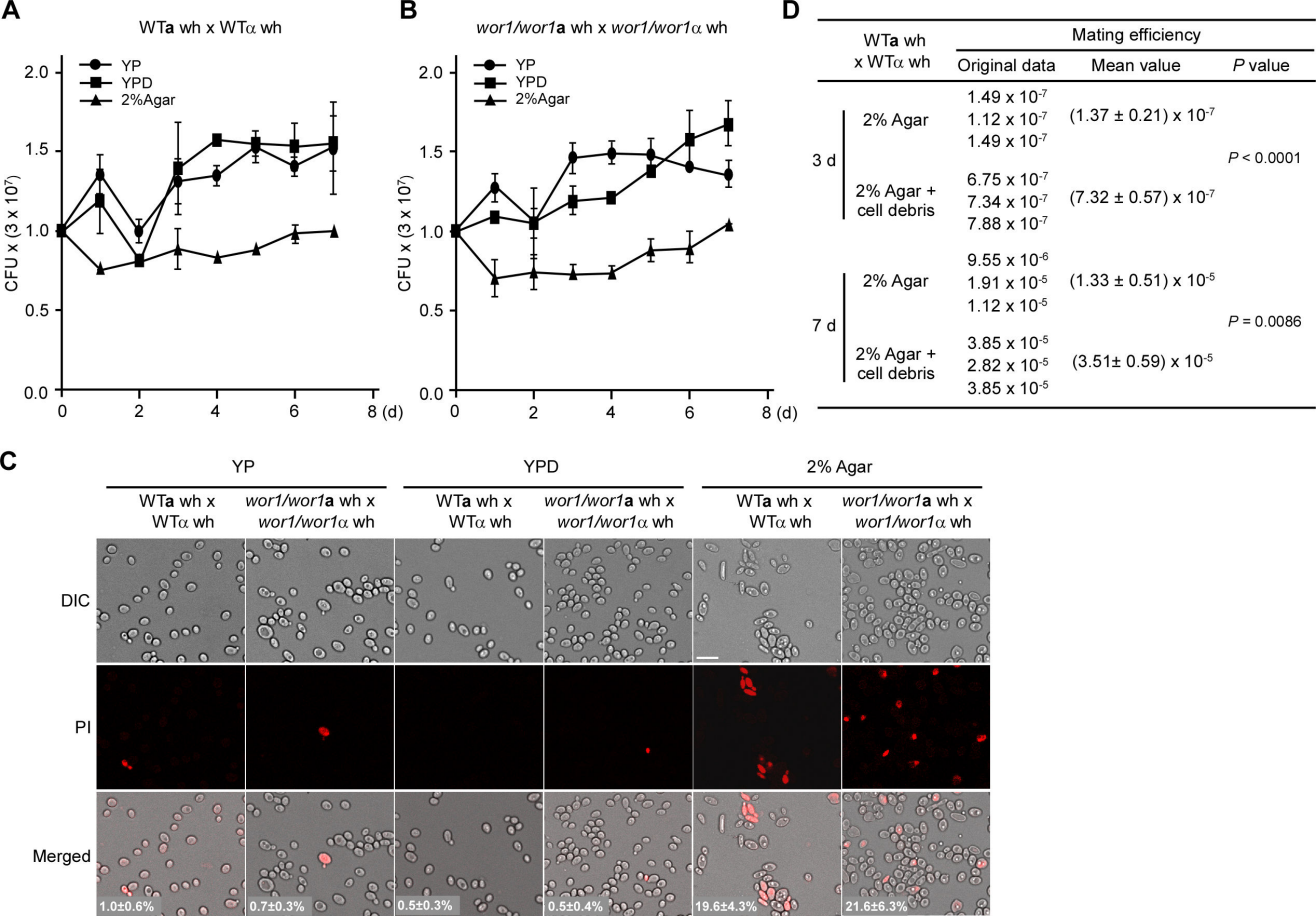
Growth curves (A and B), cell death (**C**), and mating frequencies (D) of WT**a** × WTα and *wor1/wor1***a** × *wor1/wor1*α crosses on different media. Strains used: WT**a**, LTS1024; WTα, GH1710; *wor1/wor1***a**, GH1248; *wor1/wor1*α, CAY3336. Media tested: YP, YPD, 2% agar, or 2% agar + cell debris. White **a** and α cells (3 × 10^7^ for each) were mixed, spotted onto different media, and incubated at 25°C under 25% RH. For panels A and B, cell viabilities at different time points were determined using plating assays described in Materials and Methods. Three independent experiments were performed. The result represents the average ± SD. *X*-axis, incubation time; *Y*-axis, CFU value. CFU, colony forming units; wh, white cells. (**C**) Propidium iodide (PI) staining assays were performed to indicate dead cells (red). After 5 days of incubation at 25°C under 25% RH, the mixture was collected and stained using PI. The percentages of stained cells (average ± SD) are indicated in the corresponding images. DIC, differential interference contrast; Scale bar: 10 µm. Background differences result from automatic software adjustments to fluorescence intensity. Detailed strain information is included in the supplementary materials (Table S4). (**D**) Mating frequency on 2% agar medium containing *C. albicans* debris. After 3 or 7 days of incubation at 25°C under 25% RH, the mating frequency was analyzed. Three independent experiments were performed. The result represents the average ± SD. Statistical differences were determined by two-sided unpaired Student’s *t*-test.

As essential nutrients are required for *C. albicans* white cell mating, we investigated how cells acquire nutritional components when grown on agar medium. Colony-forming unit (CFU) and cell staining assays were performed to evaluate cell viability. As shown in Fig. 3A and B, the CFU counts of both WT and *wor1/wor1* mutant crosses decreased after 1 or 2 days of incubation on 2% agar medium at 25°C, indicating that a subset of cells underwent cell death. In contrast, CFU counts increased during growth on YP or YPD media, consistent with active cell proliferation. Propidium iodide (PI) staining further confirmed that nutrient depletion-induced cell death in a subset of white cells (Fig. 3C). PI-stained cells were rarely observed during growth on YP or YPD media. Over extended incubation, CFU counts of the mating mixtures in both WT and *wor1/wor1* crosses on 2% agar medium gradually returned to their initial levels. To further verify that the nutrients released by dead cells could promote mating, we examined the mating frequency of white cells on medium plates containing *C. albicans* debris. As shown in Fig. 3D, the addition of cell debris increased the efficiency of white cell mating about sixfold at day 3 and threefold at day 7 on 2% agar medium. To further clarify the potential effect of cell debris on cell viability or promoting mating in *C. albicans*, we performed PI staining on days 3 and 7 for the cells of the WT cross (WT**a** wh × WTα wh) grown on 2% agar-only or 2% agar + cell debris medium. As shown in Fig. S2, there was no significant difference in the cell survival rates between the two groups. These results indicate that the debris has no effect on the improvement of cell viability and could function as a cue to promote white cell mating.

### Inactivation of *CST20*, *MAC1*, and mitochondrial metabolism-related genes significantly reduces the frequency of white cell mating

**Fig 4:**
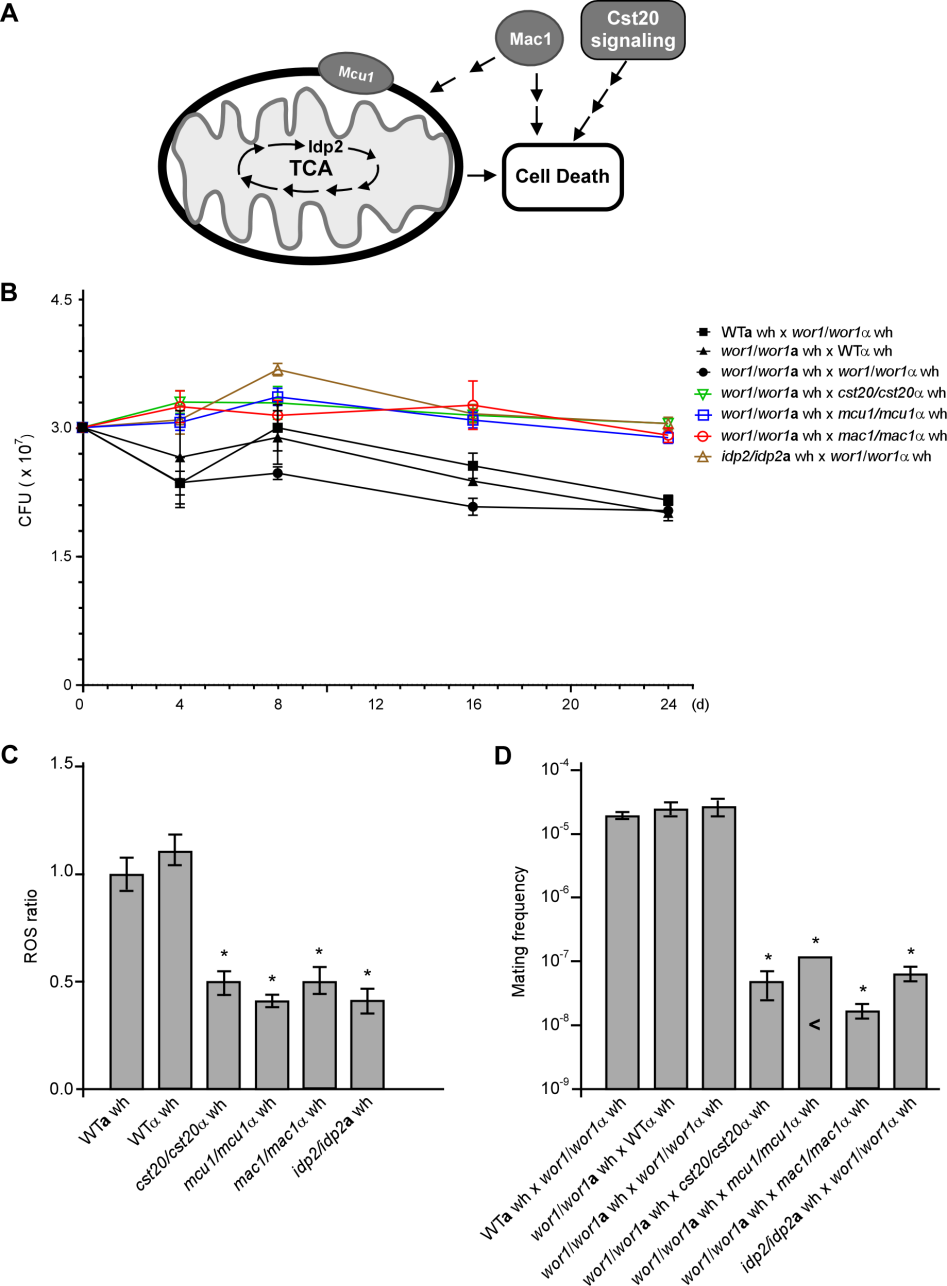
Genes associated with mitochondrial metabolism and cell death regulate nutrient depletion-induced white cell mating. (**A**) Schematic illustration of mitochondrial metabolism and cell death regulation. Mcu1 is a *Candida*-specific mitochondrial protein required for mitochondrial metabolism. Idp2 (Isocitrate dehydrogenase) is a key TCA cycle enzyme. Mac1 (a copper ion-sensing regulator) and Cst20 (a member of the p21-activated kinase family) are involved in the regulation of cell death in fungi. (**B**) Survival rates of different crosses on 2% agar medium. Cell viabilities at different time points were determined using plating assays described in Materials and Methods. Three independent experiments were performed. CFU, colony forming units; wh, white cells. (**C**) Relative ROS levels. 3 × 10^7^ white cells of the WT and deletion mutant strains were spotted onto 2% agar medium and cultured at 25°C under 25% RH for 4 days. 1 × 10^5^ cells were used for ROS level examination. Three independent experiments were performed. The result represents the average ± SD. Statistical differences were determined by two-sided unpaired Student’s *t*-test. *, *P* < 0.05. (**D**) Mating frequencies of different crosses on 2% agar medium. Three independent experiments were performed. The result represents the average ± SD. “<” indicates no mating progeny colonies observed; *, *P* < 0.05. Strains WT**a** (LTS1024), WTα (GH1710), *wor1/wor1***a** (GH1248), and *wor1/wor1*α (CAY3336) were used as controls. This figure is associated with Table S3. Detailed strain information is included in the supplemental material (Table S4).

Given that cell death in *C. albicans* is essential for nutrient acquisition and mating of surviving white cells, we hypothesized that genetic perturbation blocking cell death would affect mating frequency. To test this hypothesis, we assessed cell viability and mating frequency in mutants of *CST20*, *MAC1*, and the mitochondrial metabolism-related genes *MCU1* and *IDP2* (Fig. 4A). Cst20 is an ortholog of *Saccharomyces cerevisiae* p21-activated kinase Ste20, which is required for H_2_O_2_- and glucose-induced cell death in *S. cerevisiae* (24, 25). The copper ion-sensing transcription factor Mac1 and the mitochondrial protein Mcu1 are involved in the regulation of GlcNAc-induced cell death and filamentation in *C. albicans* (26–28). *IDP2* encodes isocitrate dehydrogenase, a key enzyme in the tricarboxylic acid cycle. We observed that the inactivation of these genes significantly increased the survival rates of *C. albicans* white cells at all time points tested under nutrient-depleted conditions (Fig. 4B). Additionally, intracellular ROS levels in the white cells of these mutants were markedly lower than those in WT controls (Fig. 4C), suggesting that nutrient depletion-induced ROS generation is at least associated with cell death.

We then performed quantitative mating assays using these mutants. As shown in Fig. 4D and Table S3, the mating frequencies of crosses between *wor1/wor1* and gene deletion mutants (c*st20*/c*st20*, *mac1/mac1*, *mcu1/mcu1*, and *idp2/idp2*) were significantly lower than those of the controls. These results indicate that nutrient depletion-induced cell death is associated with white cell mating in *C. albicans*.

## DISCUSSION

We recently demonstrated that glucose depletion enables white cells to mate at moderate frequencies by activating the mating-associated MAPK pathway (17). In this study, we show that extreme nutrient depletion induces efficient white cell mating in *C. albicans*. Nutrient depletion leads to the accumulation of intracellular ROS, which appear to drive cell death. We propose that dead cells release nutrients, supporting the mating of surviving white cells. Consistent with this hypothesis, the suppression of cell death via the inactivation of *CST20*, *MAC1*, and mitochondrial metabolism-related genes significantly reduced white cell mating frequency. These findings indicate that white cells are capable of undergoing efficient mating under certain culture conditions. Notably, the addition of cell debris increased white cell mating around sixfold and threefold on 2% agar medium at days 3 and 7, respectively (Fig. 3D). These results support that *C. albicans* cell debris is indeed able to promote white cell mating. In contrast, one previous study has reported that the mating frequency decreases around eightfold on 3% agar + cell debris compared to that on 3% agar-only medium at the later time point (day 5 vs day 7) (18). The discrepancy in mating frequencies between the previous study and our current study could be due to that the different cell types of *C. albicans* that were tested. Moreover, on 2% agar medium, white cells exhibit lower expression levels of mating-associated genes, which may correspond to their reduced mating frequency (~10^−4^) relative to opaque cells (~10^−1^) (Fig. 2). In the previous study, same-sex mating of opaque cells was examined, while in the current study, opposite-sex mating of white cells was tested. As white cells are the default state of *C. albicans* in nature and the white-to-opaque transition is unique to this fungus and its close relatives, it is plausible that opaque cells represent a more recently evolved cell type with enhanced mating capabilities; however, white cell mating may reflect a conserved sexual reproduction mechanism, similar to that observed in other fungal species such as *S. cerevisiae* and *Schizosaccharomyces pombe*.

Nutrient availability is a well-recognized signal for sexual reproduction in many fungal species (20, 21). For example, nitrogen depletion induces sexual mating in *Cryptococcus neoformans*, an opportunistic fungal pathogen responsible for severe central nervous system infections (29). Similarly, nutrient starvation combined with mating-pheromone availability triggers sexual development in *S. pombe* (30, 31). Poor nutritional conditions also promote mating in *C. albicans* opaque cells (32).

Intracellular ROS production is a conserved signal for cell death across eukaryotes (33), with mitochondrial metabolism being a primary source of ROS. Studies in *S. cerevisiae* have demonstrated the critical role of mitochondrial metabolism and the Ste20-MAP kinase pathway in regulating cell death (25). Our findings suggest that this regulatory mechanism is conserved in *C. albicans* and is associated with white cell mating (Fig. 4 and Table S3). Specifically, the inactivation of the kinase Cst20 and mitochondrial metabolism-associated genes suppressed cell death and led to a substantial reduction in white cell mating frequency. Consistent with this observation, we previously reported that mitochondrial metabolism is crucial for N-acetylglucosamine (GlcNAc)-induced cell death in *C. albicans*.

Programmed cell death, particularly in response to environmental stress, is a common phenomenon in eukaryotic single-celled organisms, which could benefit the community in multiple ways (34). For example, the altruistic phenomenon due to cell death has been observed in several microbial species including *S. cerevisiae* (25), the kinetoplastid parasites *Trypanosoma cruzi* (35), *Trypanosoma brucei rhodensiense* (36), and *Dictyostelium discoideum* (37). Our findings add the opportunistic fungal pathogen *C. albicans* to this list. Given that *C. albicans* frequently encounters environmental stresses such as nutrient depletion and oxidative stress, our findings of extreme culture conditions and cell death-promoted epigenetic switch-independent mating would provide novel insights into the biology of this major fungal pathogen.

## MATERIALS AND METHODS

### Strains and growth conditions

Detailed information on *C. albicans* strains used in this study is provided in Table S4. Strains were stored in 25% glycerol at −80°C. To recover strains from glycerol stocks, cells were scraped using a sterile tip, streaked onto YPD medium plates (20 g/L peptone, 10 g/L yeast extract, 20 g/L glucose, and 20 g/L agar, pH 7.3), and cultured at 30°C. Peptone (BD Difco 211677), yeast extract (BD Difco 212750), and agar (BD Difco 214010) were purchased from BD Biosciences (Sparks, MD, USA), and glucose (G8270) was purchased from Sigma-Aldrich (St. Louis, MO, USA).

YPD and modified Lee’s glucose or Lee’s GlcNAc medium (38) were used for routine growth of *C. albicans*. The dye phloxine B (5 µg/mL, P2759, Sigma-Aldrich) was added to solid media to stain opaque cells (39). Solid YP (20 g/L peptone, 10 g/L yeast extract, and 20 g/L agar), YPD, 2% agar (20 g/L agar), 4% agar (40 g/L agar), or 2% agar + *C. albicans* debris media were used for mating-projection induction, cell viability determination, and mating assays. To make 2% agar + *C. albicans* debris medium, around 1 × 10^10^ cells of strain SC5314 were collected from an overnight YPD culture, washed with ddH_2_O, and ground with glass beads. Agar and cell debris were mixed and resuspended in 100 mL ddH_2_O for autoclaving. For quantitative mating assays, SCD medium lacking specific nutrients (e.g., uridine [Uri], histidine [His], and/or arginine [Arg]) was used for selective growth (39). Opaque cells obtained from modified Lee’s GlcNAc medium plates containing phloxine B served as controls for mating projection formation assays.

### Strain construction

Deletion mutants were constructed using a fusion PCR strategy as described previously (40). Briefly, PCR products targeting the *CST20* locus (5′- and 3′-flanking fragments of *CST20*) were amplified from the genomic DNA of *C. albicans* strain SC5314 using primers CST20up-Fwd/CST20up-Rev and CST20down-Fwd/CST20down-Rev, respectively. Selectable marker genes (*CdARG4*, *CdHIS1*, and *CmLEU2*) were PCR amplified from plasmids pSN69, pSN52, and pSN40, respectively (40). To delete both alleles of *CST20*, the *CdHIS1* and *CmLEU2* markers flanked by 5′- and 3′-fragments of *CST20* were amplified via fusion PCR and sequentially introduced into strain SN152. A similar strategy was employed to delete the *MCU1* gene in strain SN152. For the deletion of both alleles of *IDP2*, fusion PCR products of the *CdHIS1* and *CaURA3* markers flanked by 5′- and 3′-fragments of the corresponding genes were sequentially transformed into strain GH1013. The *CdHIS1* and *CaURA3* markers were amplified from plasmids pSN52 and pGEM-URA3 (41), respectively. To construct the *MTL* homozygous strain *cst20/cst20*α, the *Apa*I/*Sac*I linearized plasmid L23.14 was transformed into the *MTL* heterozygous strain *cst20/cst20* to delete the *MTL***a** allele (39). A similar approach was used to generate the *MTL* homozygous strains *mcu1/mcu1*α and *mac1/mac1*α.

### Cell viability assay

White cells of *C. albicans* **a** and α strains (3 × 10^7^ for each) were mixed and spotted onto different media and incubated at 25°C under 25% RH for the durations indicated in the main text. A humidity-controlled cabinet was used to stabilize the 25% RH conditions (19). The media used included YP, YPD, and 2% agar. The 2% agar medium consisted of agar in water without additional nutrients to support cell growth. Cell viability at different time points was assessed by collecting cells from the various media plates and replating them onto YPD medium followed by 2 days of incubation at 30°C. CFUs were counted, and the percentage of viable cells was evaluated. Additionally, the cell viability of various deletion mutants was evaluated on 2% agar plates. Results represent the average ± SD of three independent experiments.

### PI staining assay

Propidium iodide (PI; P4170, Sigma-Aldrich) staining was performed as previously described (27). Briefly, white **a** and α cells (3 × 10^7^ for each) were mixed and spotted onto different media and incubated at 25°C under 25% RH. After 5 days of incubation, cells were collected, washed with 1× phosphate-buffered saline (PBS), and stained with PI at a final concentration of 10 µM. Stained cells were visualized using a Leica TCS SP8 DIVE FALCON microscope, and the percentage of unstained cells was calculated to determine the survival rate. Images were acquired using the same settings; however, due to automatic software adjustments to fluorescence intensity, background appearance may vary. Modifications were made post-acquisition.

### Assay of pheromone-induced mating projection formation

A chemically synthesized 14-mer α-pheromone peptide (GFRLTNFGYFEPGK) was used to induce mating projections in *C. albicans* as previously described (16). White or opaque cells (1.5 × 10^7^) of *C. albicans* were spotted onto 2% agar, YP, or YPD media and incubated at 25°C under 25% RH. Three microliters of α-pheromone solution (200 µM) was added to each spot every 24 h (at 24, 48, 72, 96, and 120 h). After 7 days of incubation, cells were collected for microscopy analysis, and the percentages of cells forming mating projections were calculated.

### Quantitative mating assay

Quantitative mating assays were performed as previously described, with minor modifications (17). Briefly, white cells of *C. albicans* **a** and α mating partners (3 × 10^7^ for each) were collected from Lee’s glucose medium plates, mixed, and spotted onto various media, including YP, YPD, 2% agar, and 4% agar. After 7 days of incubation at 25°C under 25% RH, mating mixtures were resuspended, diluted, and plated onto SCD medium plates lacking specific nutrients, depending on the auxotrophic requirements of the mating partners. Mating frequencies were calculated as follows (42). Mating frequency = (number of colonies on double media)/(number of colonies on single dropout media). Three biological replicates were conducted for each mating cross.

### RNA extraction and quantitative real-time PCR (qRT-PCR) assays

RNA extraction and qRT-PCR assays were performed as previously described, with modifications (18). Briefly, cells of the *wor1/wor1***a** (GH1248) strain were harvested from Lee’s glucose medium plates, washed, and resuspended in ddH_2_O at a concentration of 3 × 10^9^ cells/mL. Subsequently, 10 µL of cells was spotted onto various media, including YP, YPD, and 2% agar. After 5 days of incubation at 25°C under 25% RH, cells were harvested and washed with PBS. Total RNA was extracted using the GeneJET RNA Purification Kit (K0732, Thermo Scientific, Waltham, USA). RNA quantity was determined using an ultraviolet spectrophotometer (Thermo Fisher, China). For qRT-PCR, 1 µg of RNA was reverse-transcribed into complementary DNA (cDNA) using ReverAid H Minus Reverse Transcriptase (EP0451, Thermo Scientific). qRT-PCR was performed using the CFX96 Real-Time PCR Detection System (Bio-Rad, Hercules, USA) with SYBR green master mix (QPS-201, TOYOBO, Osaka, Japan) and analyzed using CFX96 Manager 3.1 Software (Bio-Rad). Gene expression levels were normalized to the expression of the *ACT1* gene. Three biological replicates were conducted for each growth condition.

### Assays of white-opaque switching

White-opaque switching assays were performed as previously described (39). White cells of each strain were collected from Lee’s glucose medium plates, washed, and resuspended in ddH_2_O at a concentration of 3 × 10^9^ cells/mL. Subsequently, 5 µL of cells was spotted onto 2% and 4% agar medium plates. After 7 days of incubation at 25°C under 25% RH, cells were harvested, washed, and plated on Lee’s glucose medium to calculate white-opaque switching frequency. White-opaque switching frequency (%) was determined as: White-opaque switching frequency (%) = (number of opaque colonies + colonies with opaque sections/total number of colonies) × 100%.

### Determination of ROS levels

White cells (3 × 10^7^) were spotted onto 2% agar medium and incubated at 25°C under 25% RH for 4 days. Cells were then scraped from the medium plates, washed, and resuspended in PBS at a final concentration of 1 × 10^5^ cells/mL. Briefly, 1 µL of the DCFH-DA probe (Beyotime, Shanghai, China) was added to the cell suspension, followed by incubation at 37°C for 40 min in the dark. Fluorescence intensity was measured using an ELISA reader (Cytation 3, BioTek Instruments Inc., Winooski, VT, USA) with excitation and emission wavelengths set at 488 and 525 nm, respectively. Five independent experiments were performed for each strain, and fluorescence values were normalized to cell number. The ratio of the fluorescence value of each mutant strain to that of the WT strain was calculated.

## SUPPLEMENTAL MATERIAL

Supplemental Material msphere.00291-25-s0001.docx Figures S1 to S3; Tables S1 to S5.

ASM does not own the copyrights to Supplemental Material that may be linked to, or accessed through, an article. The authors have granted ASM a non-exclusive, world-wide license to publish the Supplemental Material files. Please contact the corresponding author directly for reuse.
