# Supplementary material for: An extremely poor nutritional condition enables efficient white cell mating in Candida albicans
Source: mSphere. 2025 Jun 5;10(6):e00291-25. doi: 10.1128/msphere.00291-25 (PMC12188724; doi:10.1128/msphere.00291-25)
Supplement: Supplemental Material — Figures S1 to S3; Tables S1 to S5. [file msphere.00291-25-s0001.docx]

**
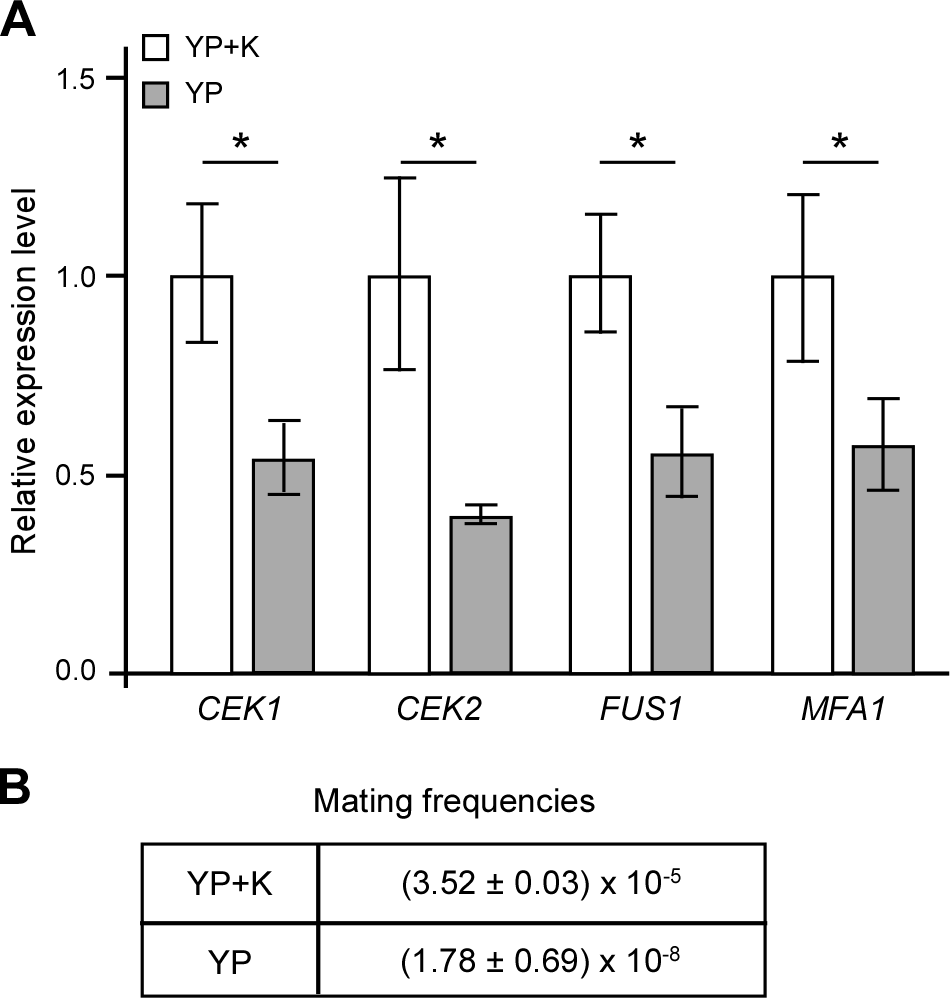
**

**Fig S1. The expression of mating-associated genes (A) and mating frequencies (B) of white cells on YP and YP+K media.** Strains used: WT**a**, LTS1024; WTα, GH1710; *wor1/wor1***a**, GH1248. (A) Relative expression levels of mating-associated genes. Approximately 3 ×10^7^ cells of the *wor1/wor1***a** (GH1248) were spotted onto YP and YP+K media, and incubated at 25 °C under 25% RH for 5 days. Statistical differences were determined by two-sided unpaired Student’s *t*-test. *, P < 0.05. (B) Mating frequencies. Approximately 3 ×10^7^ cells of the WT**a** white (LTS1024) and WTα white (GH1710) were mixed and spotted onto YP and YP+K media, and incubated at 25 °C under 25% RH for 7 days. Three independent experiments were performed. The result represents the average ± standard deviation (SD).


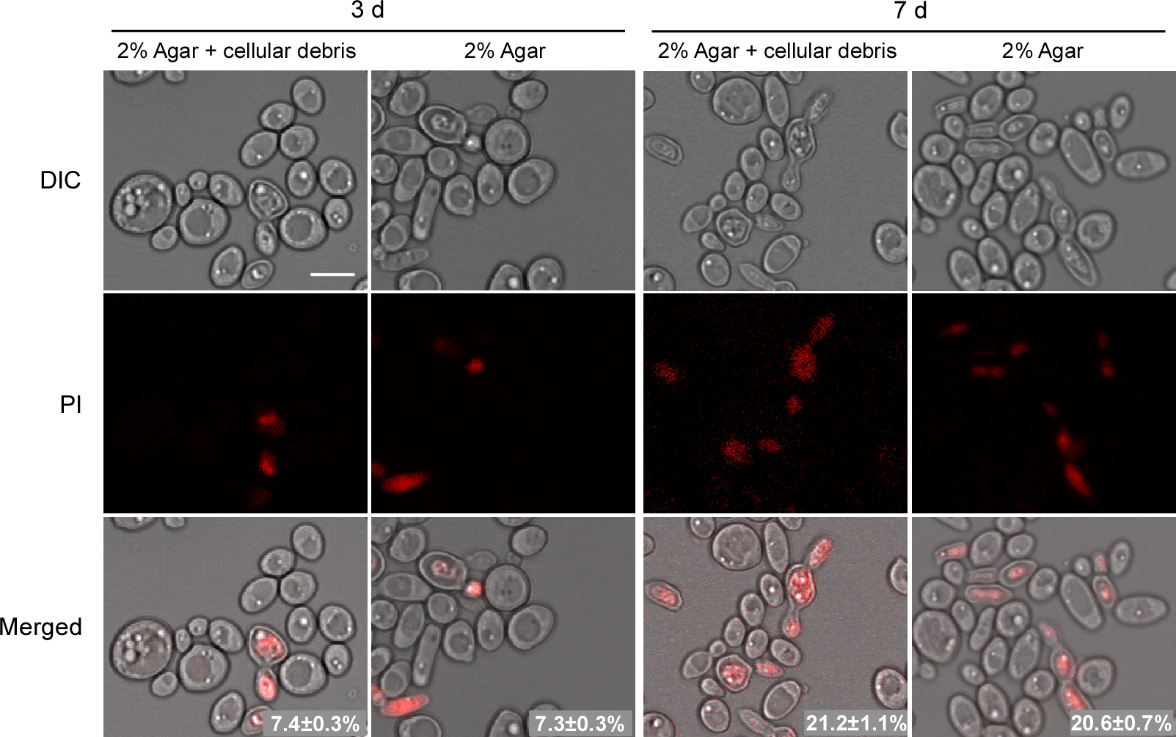


**Fig S2. PI staining assays of the mating mixtures of WTa x WTα cross on 2% agar + *C. albicans* debris medium.** Propidium iodide (PI) staining assays were performed to indicate dead cells (red). After 3 or 7 days of incubation at 25 °C under 25% RH, the mixture was collected and stained using PI. The percentages of stained cells (average ± SD) are indicated in the corresponding images. DIC, differential interference contrast; Scale bar: 10 μm.


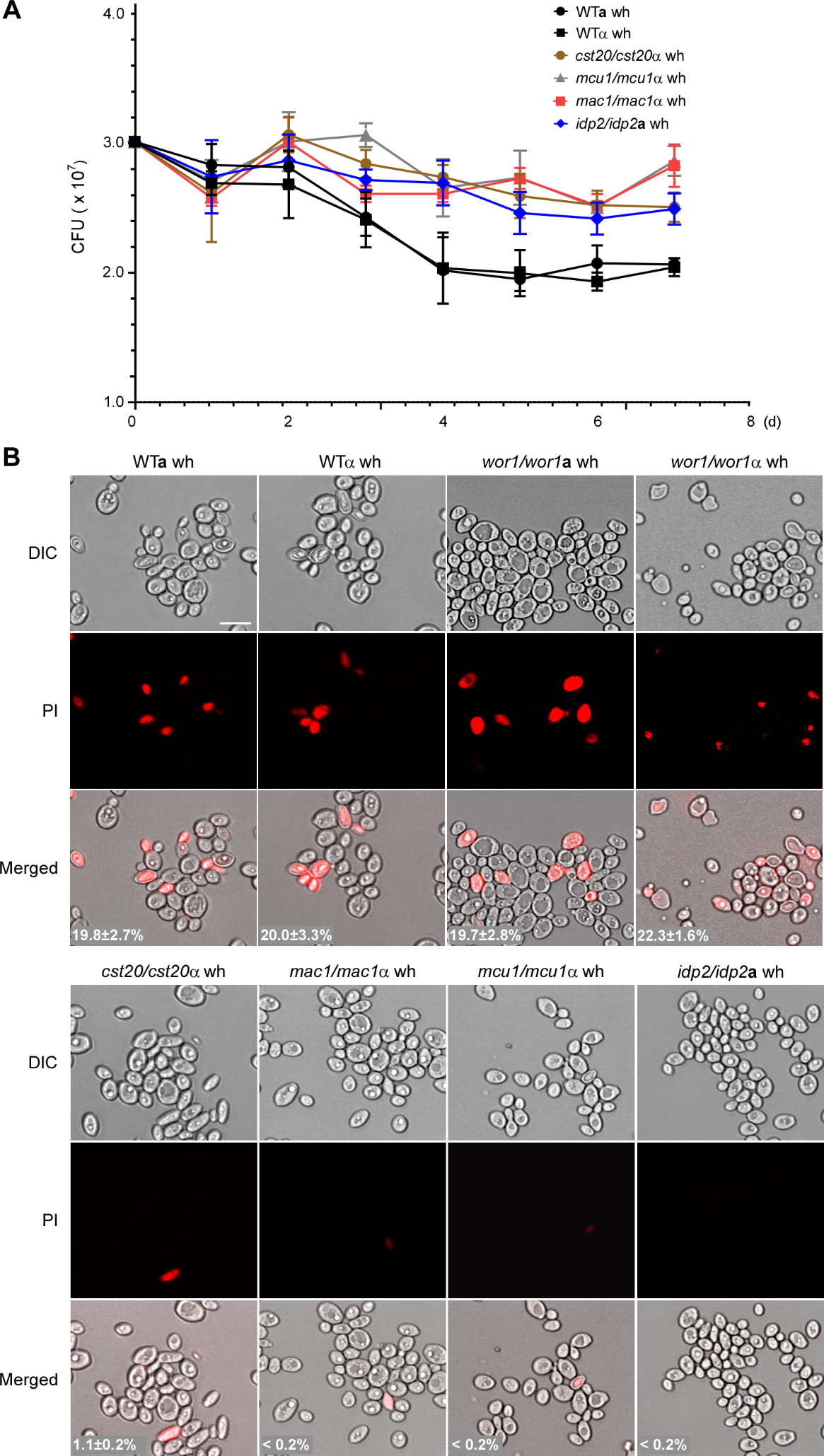


**Fig S3. Survival curves (A) and cell death (B) of different mutant strains on** **2% agar medium.** Two WT stains and four gene deletion mutants were examined. Strains used: WT**a**, LTS1024; WTα, GH1710. (A) Cell viabilities at different time points were determined using plating assays described in the methods section. Three independent experiments were performed. wh, white cells. CFU: Colony forming units. (B) Propidium iodide (PI) staining assays were performed to indicate dead cells (red). After 5 days of incubation on 2% agar medium at 25 °C under 25% RH, the cells of each mutant strain were collected and stained using PI. The percentages of stained cells (average ± SD) are indicated in the corresponding images. DIC, differential interference contrast; Scale bar: 10 μm.

**Table S1. Mating freqencies of the WT and *wor1/wor1* mutant strains of *C. albicans* on different culture media.**

| **Mating mixtures** | **YP** | **YPD** | **2% Agar** | **4% Agar** |
| --- | --- | --- | --- | --- |
| WT**a** wh × WTα wh | (4.31 ± 3.73) × 10^-8^ | (2.14 ±0.59) × 10^-7^ | (1.06 ± 0.24) × 10^-4^ | (1.87 ±0.61) × 10^-4^ |
| *wor1/wor1***a** × *wor1/wor1*α | (1.12 ± 1.23) × 10^-8^ | (4.34 ± 0.61) × 10^-8^ | (3.93 ± 0.40) × 10^-5^ | (5.53 ± 1.03) × 10^-5^ |

**Notes:** White cells of the **a** and α mating partners (3 ×10^7^ cells for each) were mixed, spotted onto different medium plates, and incubated at 25^°^C under 25% relative humidity (RH). YPD, YP, 2% agar, and 4% agar media were examined. After seven days of incubation, the mating mixtures were plated onto synthetic complete dextrose (SCD) media lacking corresponding nutrients to determine mating frequencies. wh, white cells. Strains used: WT**a**, LTS1024; WTα, GH1710; *wor1/wor1***a**, GH1248; and *wor1/wor1*α, CAY3336. Three independent experiments were performed. This Table is associated with **Figure 1**.

**Table S2. White-opaque switching of different deletion mutants under nutrient depletion conditions.**

| Strain |  | White-opaque switching frequencies | | | | |
| --- | --- | --- | --- | --- | --- | --- |
|  |  | 2% Agar | |  | 4% Agar | |
|  |  | Total colonies | % op colonies |  | Total colonies | % op colonies |
| WT**a** wh |  | 474 | < 0.21 |  | 593 | < 0.17 |
| WTα wh |  | 492 | < 0.20 |  | 469 | < 0.21 |
| *cst20/cst20*α wh |  | 854 | < 0.12 |  | 997 | < 0.10 |
| *mcu1/mcu1*α wh |  | 301 | < 0.33 |  | 323 | < 0.31 |
| *mac1/mac1*α wh |  | 758 | < 0.13 |  | 624 | < 0.16 |
| *idp2/idp2***a** wh |  | 433 | < 0.23 |  | 516 | < 0.19 |

**Notes:** White cells of each strain were collected from Lee’s glucose medium plates, and then washed and resuspended in ddH_2_O at a concentration of 3 × 10^9^ cells/mL. 5 μL of cells were spotted onto both 2% agar and 4% agar medium plates. After 7 days of incubation at 25 ^o^C under 25% relative humidity (RH), cells were harvested, washed, and plated on Lee’s glucose medium for calculating white-opaque switching frequencies. wh, white cells. Strains used: WT**a**, LTS1024; WTα, GH1710. Detailed strain information is presented in **Table S4**. Three independent experiments were performed. “<” indicates no opaque colonies observed.

| Mating mixtures | 2% Agar |
| --- | --- |
| WT**a** wh × *wor1/wor1*α wh | (1.91 ± 0.18) × 10^-5^ |
| *wor1/wor1***a** wh × WTα wh | (2.48 ± 0.62) × 10^-5^ |
| *wor1/wor1***a** wh × *wor1/wor1*α wh | (2.72 ± 0.84) × 10^-5^ |
| *wor1/wor1***a** wh × *cst20/cst20*α wh | (4.73 ± 2.31) × 10^-8^ |
| *wor1/wor1***a** wh × *mcu1/mcu1*α wh | < 1.19 × 10^-7^ |
| *wor1/wor1***a** wh × *mac1/mac1*α wh | (1.72 ± 0.46) × 10^-8^ |
| *idp2/idp2***a** wh × *wor1/wor1*α wh | (6.43 ± 1.87) × 10^-8^ |

**Table S3. Mating frequencies of different deletion mutants under nutrient depletion conditions.**

**Notes:** White cells of the **a** and α mating partners (3 ×10^7^ cells for each) were mixed, spotted onto 2% agar medium, and incubated at 25^°^C under 25% relative humidity (RH). After seven days of incubation, the mating mixtures were plated onto synthetic complete dextrose (SCD) media lacking corresponding nutrients to determine mating frequencies. wh, white cells. Strains used: WT**a**, LTS1024; WTα, GH1710; *wor1/wor1***a**, GH1248; and *wor1/wor1*α, CAY3336. Detailed strain information is presented in **Table S4**. Three independent experiments were performed. The result represents the average ± standard deviation (SD). “<” indicates no mating progeny colonies observed. Statistical differences were determined by two-sided unpaired Student’s *t*-test. *, P < 0.05. This table is associated with **Figure 4**.

**Table S4 Strains used in this study.**

| **Strain name** | **Parent strain** | **Genotype** | **Reference** |
| --- | --- | --- | --- |
| SC5314 |  | *MTL***a***/α* Clinical isolate | [1] |
| CAI4 | SC5314 | *MTL***a***/α ura3::imm434/ura3::imm434* | [1] |
| SN152 | CAI4 | As CAI4, but *URA3/ura3::imm434 iro1::IRO1/iro1::imm434 his1::hisG/his1::hisG leu2/leu2 arg4/arg4* | [2] |
| GH1013 | CAI4 | *MTL***a**/**a** *ura3::imm434*/*ura3::imm434* *his1::hisG*/*his1::hisG* *arg4::hisG*/*arg4::hisG* | [3] |
| WT **a** (LTS1024) | GH1013 | As GH1013, but *his1::hisG* /*his1::hisG*::*HIS1 arg4::hisG* /*arg4::hisG::ARG4* | [4] |
| SN250 | CAI4 | As CAI4, but *ura3::imm434::URA3-IRO1/ura3::imm434 arg4::hisG/arg4::hisG his1::hisG/his1::hisG leu2::hisG::CdHIS1/leu2::hisG::CmLEU2* | [5] |
| WT α (GH1710) | SN250 | As SN250, but *MTLα/mtl***a***::FRT* | [4] |
| *wor1*/*wor1* **a** (GH1248) | GH1013 | As GH1013, but *wor1::ARG4*/*wor1::HIS1* | [6] |
| *wor1/wor1* α (CAY3336) |  | *MTLα/α*, *ura3::imm434::URA3/ura3::imm434*  *iro1::IRO1/iro1::imm434 his1::hisG/his1::hisG*  *leu2/leu2, wor1::FRT/wor1::FRT* | [7] |
| *cst20/cst20* | SN152 | As SN152, but *cst20::HIS1/cst20::LEU2* | This study |
| *cst20/cst20* α | *cst20/cst20* | As *cst20/cst20*, but *MTLα/mtl***a***::FRT-SAT1-FRT* | This study |
| *mcu1/mcu1* | SN152 | As SN152, but *mcu1::HIS1/mcu1::LEU2* | This study |
| *mcu1/mcu1* α | *mcu1/mcu1* | As *mcu1/mcu1*, but *MTLα/mtl***a***::FRT-SAT1-FRT* | This study |
| *mac1/mac1* | SN152 | As SN152, but *mac1::LEU2 /mac1::HIS1* | [8] |
| *mac1/mac1* α | *mac1/mac1* | As *mac1/mac1*, but *MTLα/mtl***a***::FRT-SAT1-FRT* | This study |
| *idp2/idp2* **a** | GH1013 | As GH1013, but *idp2::HIS1* /*idp2::URA3* | This study |

**References:**

1. Fonzi WA, Irwin MY. Isogenic strain construction and gene mapping in *Candida albicans*. *Genetics* 134(3):717-728 (1993).
2. Noble SM, Johnson AD. Strains and strategies for large-scale gene deletion studies of the diploid human fungal pathogen *Candida albicans*. Eukaryot Cell 4: 298-309 (2005).
3. Huang G, Srikantha T, Sahni N, Yi S, Soll DR. CO_2_ regulates white-to-opaque switching in *Candida albicans*. Curr Biol 19: 330-334 (2009).
4. Guan G, Tao L, Li C, Xu M, Liu L, Bennett RJ, Huang G. Glucose depletion enables *Candida albicans* mating independently of the epigenetic white-opaque switch. Nat Commun 14(1):2067 (2023).
5. Noble SM, French S, Kohn LA, Chen V, Johnson AD. Systematic screens of a *Candida albicans* homozygous deletion library decouple morphogenetic switching and pathogenicity. Nat genet 42: 590-598 (2010).
6. Huang G, Wang H, Chou S, Nie X, Chen J, Liu H. Bistable expression of *WOR1*, a master regulator of white-opaque switching in *Candida albicans*. Proc Natl Acad Sci U S A 103: 12813-12818 (2006).
7. Scaduto CM, Kabrawala S, Thomson GJ, Scheving W, Ly A, Anderson MZ, Whiteway M, Bennett RJ. Epigenetic control of pheromone MAPK signaling determines sexual fecundity in *Candida albicans*. Proc Natl Acad Sci U S A 114: 13780-13785 (2017).
8. Du H, Guan G, Li X, Gulati M, Tao L, Cao C, Johnson AD, Nobile CJ, Huang G. N-acetylglucosamine-induced cell death in *Candida albicans* and its implications for adaptive mechanisms of nutrient sensing in yeasts. mBio. 6(5):e01376-15 (2015).

**Table S5 Primers used in this study.**

| **Name** | **Sequence (5’ to 3’)** | **Purpose** |
| --- | --- | --- |
| CST20up-Fwd | TCATTTCACCACTCACTCAAC | Knockout of *CST20* |
| CST20up-Rev | CACGGCGCGCCTAGCAGCGGAAAGAGTCAACAAAAGTGCAG |  |
| LT2 | CCGCTGCTAGGCGCGCCGTGaccagtgtgatggatatctgc |  |
| LT5 | GCAGGGATGCGGCCGCTGACagctcggatccactagtaacg |  |
| CST20down-Fwd | GTCAGCGGCCGCATCCCTGCTGAATGGTGGAGTTATCCTAG |  |
| Cst20down-Rev | TACGGAAAACCAATTACAGC |  |
| CST20-check Fwd | TAATCAGAGAGAGAAAGAGAG | Verification of the *cst20/cst20* mutant |
| HIS1-5-detect | ATTAGATACGTTGGTGGTTC |  |
| HIS1-3-detect | AACACAACTGCACAATCTGG |  |
| LEU2-5-detect | AGAATTCCCAACTTTGTCTG |  |
| LEU2-3-detect | AAACTTTGAACCCGGCTGCG |  |
| CST20-check Rev | TGCATCATTAGGACGAGCATC |  |
| CST20 ORF Fwd | TGGACCTGAAGTTGAATCAAC |  |
| CST20 ORF Rev | TTGGTGTTGTAAGGACAATG |  |
| MCU1up-Fwd | CGAATCACAATCATCTCCAG | Knockout of *MCU1* |
| MCU1up-Rev | CACGGCGCGCCTAGCAGCGGGTTTGGCTTCTTGTAATAGTG |  |
| MCU1down-Fwd | GTCAGCGGCCGCATCCCTGCCAAGTGGAGTAACGAGTATG |  |
| MCU1down-Rev | CAACCTTCCATTTATCCGTCC |  |
| uMCU1-check Fwd | ATCAGAAGTAAGTCCACAGTAG | Verification of the *mcu1/mcu1* mutant |
| MCU1-check Rev | GGATCTTGTTTGTGGTTGAAG |  |
| MCU1 ORF Fwd | TACCAGGCCAAGAACCAAGTG |  |
| MCU1 ORF Rev | CATCTCTAACATTTCTAACCAC |  |
| IDP2up-Fwd | TGTATGTGTGTGGATGTGG | Knockout of *IDP2* |
| IDP2up-Rev | CACGGCGCGCCTAGCAGCGGTGGGATTCTTGATTTCCTC |  |
| IDP2down-Fwd | GTCAGCGGCCGCATCCCTGCAACCCGGTCCAACTCTTTC |  |
| IDP2down-Rev | TCATCCTGTTGTGCTCCTTG |  |
| IDP2-check Fwd | TTGATTAGCGATTCACGGAG | Verification of the *idp2/idp2* mutant |
| ARG4-5-detect | TGCATTGACTACAGTGGAAC |  |
| ARG4-3-detect | ATCATGCCATTCTTGTCTG |  |
| IDP2-check Rev | AGAAAAGCAATGCCCTTAAC |  |
| IDP2 ORF Fwd | TCACCACTGATGCTGCCAATG |  |
| IDP2 ORF Rev | TCTAGTGACAGTACCGTGAG |  |
| LT1878 | TCAACAGCAACATCAACAAC | *CEK1* RT-PCR |
| LT1879 | TTGAATGAAACTTGACGAGG |  |
| LT1880 | ATTCTGTCTTCGTACGTTACG | *CEK2* RT-PCR |
| LT1881 | GATATTATGCAAATCCAGAGG |  |
| LT662 | TAGCAAAAGCTCTCCAAATG | *FUS1* RT-PCR |
| LT663 | TGCGATGTAGATGGTACTTTC |  |
| LT549 | ATGGCTGCTCAACAACAATC | *MFA1* RT-PCR |
| LT550 | AACAGAACAAGTGGAACAGC |  |
| LT434 | TTTCCTACGTGTACTTGTGC | *ACT1* RT-PCR |
| LT435 | TATAGTTGTGTGCACTGAGC |  |
